# Automatic uncovering of patient primary concerns in portal messages using a fusion framework of pretrained language models

**DOI:** 10.1093/jamia/ocae144

**Published:** 2024-06-27

**Authors:** Yang Ren, Yuqi Wu, Jungwei W Fan, Aditya Khurana, Sunyang Fu, Dezhi Wu, Hongfang Liu, Ming Huang

**Affiliations:** Department of Computer Science and Engineering, University of South Carolina, Columbia, SC 29208, United States; Department of Artificial Intelligence and Informatics, Mayo Clinic, Rochester, MN 55905, United States; Department of Artificial Intelligence and Informatics, Mayo Clinic, Rochester, MN 55905, United States; Department of Radiation Oncology, Mayo Clinic, Rochester, MN 55905, United States; Department of Health Data Science and Artificial Intelligence, University of Texas Health Science Center at Houston, Houston, TX 77030, United States; Department of Integrated Information Technology, University of South Carolina, Columbia, SC 29208, United States; Department of Health Data Science and Artificial Intelligence, University of Texas Health Science Center at Houston, Houston, TX 77030, United States; Department of Artificial Intelligence and Informatics, Mayo Clinic, Rochester, MN 55905, United States; Department of Health Data Science and Artificial Intelligence, University of Texas Health Science Center at Houston, Houston, TX 77030, United States

**Keywords:** patient portal message, patient concern, text classification, BERT, pretrained language model, natural language processing, deep learning, patient centered care

## Abstract

**Objectives:**

The surge in patient portal messages (PPMs) with increasing needs and workloads for efficient PPM triage in healthcare settings has spurred the exploration of AI-driven solutions to streamline the healthcare workflow processes, ensuring timely responses to patients to satisfy their healthcare needs. However, there has been less focus on isolating and understanding patient primary concerns in PPMs—a practice which holds the potential to yield more nuanced insights and enhances the quality of healthcare delivery and patient-centered care.

**Materials and Methods:**

We propose a fusion framework to leverage pretrained language models (LMs) with different language advantages *via* a Convolution Neural Network for precise identification of patient primary concerns *via* multi-class classification. We examined 3 traditional machine learning models, 9 BERT-based language models, 6 fusion models, and 2 ensemble models.

**Results:**

The outcomes of our experimentation underscore the superior performance achieved by BERT-based models in comparison to traditional machine learning models. Remarkably, our fusion model emerges as the top-performing solution, delivering a notably improved accuracy score of 77.67 ± 2.74% and an *F*1 score of 74.37 ± 3.70% in macro-average.

**Discussion:**

This study highlights the feasibility and effectiveness of multi-class classification for patient primary concern detection and the proposed fusion framework for enhancing primary concern detection.

**Conclusions:**

The use of multi-class classification enhanced by a fusion of multiple pretrained LMs not only improves the accuracy and efficiency of patient primary concern identification in PPMs but also aids in managing the rising volume of PPMs in healthcare, ensuring critical patient communications are addressed promptly and accurately.

## Introduction

The patient portal is a cutting-edge digital health platform that has revolutionized how healthcare providers may communicate and engage with patients by offering a convenient, efficient, and secure means of accessing and managing their health information, such as their medical records, lab results, and prescriptions.^1^ Patients can also communicate with their healthcare providers through secure messaging features, resulting in a seamless patient experience that promotes greater patient engagement, increased transparency, and improved health outcomes.^2^ Thus, the patient portal system has become essential for patients to access their health information and gain support from their providers between visits, especially for those with chronic conditions and ongoing medical treatment,^3^ with reduced administrative costs, improved patient satisfaction, and patient-centered care.^4^

Among the features provided by patient portals, patient portal messages (PPMs) have emerged as a vital channel of communication between patients and healthcare providers between medical visits.^5^ As healthcare organizations increasingly adopt digital technologies to improve patient engagement and care, patient portals have gained popularity and thus the volume of PPMs has risen significantly in recent years.^6^^,^^7^ While this surge in communication has the potential to improve patient care and satisfaction, it has also introduced challenges related to efficient management and timely responses to portal messages.^8^ The task of handling PPM triage, which involves categorizing and prioritizing messages for appropriate responses, has become a critical responsibility for healthcare teams. Traditionally, the PPM triage has been performed manually by healthcare providers or administrative staff, turning out to be time-consuming and costly.^9^ The growth of PPM volume has significantly increased the workload of PPM triage, leading to potential overburden of healthcare providers or administrative staff, delays in patient care, and potentially increased risks to patient safety.^10^

To address this challenge, automated solutions enabled by artificial intelligence (AI) and natural language processing (NLP) have garnered attention for their potential to process PPMs. The automated systems can streamline PPM triage, reducing the burden on healthcare providers and administrative staff for swift responses and optimal patient care.^11^^,^^12^ Previous research has showcased the promise of NLP and machine learning in message triage, largely recognizing the existence of specific communication topics, such as patient concerns and needs, through multiple binary classifiers.^13–15^ For example, in 2015, a research study utilized binary classification techniques with traditional rule-based (RB) models and machine learning algorithms such as Naïve Bayesian (NB), logistic regression (LR), and random forest (RF) to parse the content of 1000 PPMs, aiming to discern patient concerns and needs and other communication topics.^15^ Building upon these findings, in 2017, the same research group expanded their analysis to encompass 3253 PPMs. Their extended study corroborated the initial results, revealing that RB, LR, and RF models exhibited superior efficacy, with an area under the receiver operating characteristic (ROC) curve (AUC) ranging from 0.84 to 0.93.^14^ Besides traditional machine learning models, deep learning models were also applied to the PPMs binary classification task. In the same year, a research team from the same institute utilized a convolutional neural network (CNN) model with Doc2Vec embedding^16^ for automated binary classification of portal message content.^13^ The CNN model achieved state-of-the-art performance with AUC scores between 0.92 and 0.94 and *F*1 scores ranging from 0.50 to 0.88. However, there has been comparatively limited attention given to triage of PPMs with multi-class classification,^17–19^ especially isolating and comprehending the primary concerns of patients within PPMs. Moreover, despite pretrained language models (LMs) dominating in the fields of machine learning and pushing the state-of-the-art of NLP,^20^ the application of pretrained LMs remains largely unexplored for classifying PPMs.^18^^,^^19^ In our previous work, we explored multiple pretrained LMs for PPM triage, and our results show the effectiveness of pretrained LMs on multi-class classification of PPMs.^18^

In recent text classification studies, the trend of fusing diverse deep learning models has gained prominence due to its superior classification power. This technique merges various model strengths, enhancing text representation and classification capability.^21^^,^^22^ For example, CNNs and recurrent neural networks (RNNs) are 2 popular deep learning architectures and exhibit distinct strengths in understanding natural language.^23^ RNNs excel at capturing temporal and contextual features, particularly the long-term dependencies between entities, while CNNs have the strength in capturing multiple aspects of text data by hierarchically distilling local features through convolutional filters and then combining them into higher-level semantic features.^24^ Integrating CNNs and RNNs for text classification has been shown to enhance performance, some of which achieved state-of-the-art results on multiple datasets upon their publication.^25^ Notable models that combine these approaches with various strategies include Recurrent Convolutional Neural Networks in 2015,^26^ the C-LSTM Neural Network in 2015,^27^ the attention-based combination model of CNN and RNN (Att-RCNN and CRAN) in 2019.^28^ Subsequently, transformer-based deep learning models, like the Bidirectional Encoder Representations from Transformers (BERT), have further advanced the state-of-the-art in NLP.^29^ Extensive research studies have explored combining transformers with CNNs (and/or RNNs) for text classification, showcasing models such as BERT-CNN, SBERT-CNN, and BERT-CNN-RNN that have achieved improved performance.^30–33^ In these studies, BERT-based models serve to generate contextual representations of semantic information within the text, while CNNs (and/or RNNs) are employed to transform these embeddings further, identifying meaningful patterns for classification purposes. These fusion methods demonstrate the benefits of combining different models’ advantages for text classification. Given the complexity of patient communication, an approach by combining multiple models enables more precise predictions of patient primary concerns compared to single-model techniques. Nonetheless, there has been limited exploration into integrating various LMs with diverse strengths for text classification tasks such as patient primary concern detection and PPM triage.

Thus, in this study, we build upon our initial exploration of using pretrained LMs for PPM classification around patient primary concerns, by further developing a fusion framework to enhance the classification performance. Uncovering patient primary concerns provides a deeper understanding of patient needs and enables more personalized and responsive healthcare interventions that can significantly enhance patient satisfaction and outcomes. We will harness the power of pretrained LMs to accurately identify patient primary concerns within PPMs for triage. As such, we propose a fusion framework that combines the strengths of multiple pretrained LMs, for each possessing distinct language processing capabilities with a CNN. The proposed fusion framework seeks to enhance the performance of the PPM triage by effectively capturing the nuances of patient primary concerns and delivering precise categorizations. In addition, we assess the performance of various traditional machine learning models and deep learning methods, particularly BERT-based LMs. By evaluating 3 traditional models and 9 BERT-based models—including generic, domain-specific, and source-specific variants—we compare their capabilities for PPM classification based on patient primary concerns. Our fusion model then takes center stage, revealing its capacity to achieve superior accuracy and *F*1 scores compared to individual learning models.

This study not only advances the effectiveness of PPM triage and patient primary concern discovery but also holds broader implications for text classification tasks. By harnessing the potential of different pretrained LMs, our approach addresses the mounting challenges posed by escalating PPM volumes, with a promise that patient concerns are promptly and accurately addressed within the dynamic landscape of healthcare communication. Efficient triage of PPMs holds the potential to optimize healthcare workflows and facilitate the judicious distribution of healthcare resources. Furthermore, the elucidation of patient concerns within these communications not only underscores the value of patient-centered care but also contributes to the enhancement of overall care quality by ensuring that patients’ voices are not only heard but also promptly addressed.

## Methods

### Study dataset

The dataset we acquired in this study includes 2239 PPMs generated by patients during October 16 and December 23, 2018,^34^ which were collected from the Unified Data Platform (UDP) at Mayo Clinic, Rochester, MN, with the approval of the Institutional Review Board (IRB# 18-009868). We designed a categorization strategy by taking into account the diversity of patient concerns, the frequency of these concerns, and their importance in patient care. Our strategy led to the identification of 4 categories of patient concerns including “Active Symptom Concerns (A),” “Prescription Concerns (P),” “Logistical Concerns (L),” and “Other Concerns/General Updates (U).” “Active Symptom Concerns” is dedicated to capturing patient concerns regarding current or ongoing symptoms that are actively affecting the patient. “Prescription Concerns” embodies the discussions centered around pharmacological and other treatments. “Logistical Concerns” captures the spectrum of patient communications that deal with the operational and administrative facets of patient interaction with healthcare services such as scheduling appointments, billing inquiries, and insurance matters. “Other Concerns/General Updates,” a catch-all type, includes general health updates, miscellaneous inquiries, and any other concerns that patients may express which do not specifically pertain to active symptoms, prescriptions, or logistics. Sequentially, our research team annotated the PPMs and categorized them into the 4 predominant classes using an annotation guide developed based on domain expertise from medical professionals such as physicians, nurses, informaticists, and electronic medical assistants.^34^ The inter-annotator agreement (IAA) was assessed with the *F*1 score, achieving a value of 76.92%. This indicates a good agreement among annotators, highlighting their consistent interpretations despite the complexity and possible ambiguities present in the message categorization. The annotated corpus shows a well distribution of PPMs across the 4 categories of patient primary concerns: category “A” accounted for 44.3% with 991 PPMs, category “P” comprised 13.4% with 301 PPMs, category “L” made up 14.1% with 315 PPMs, and category “U” constituted 28.2% with 632 PPMs. The detailed definition and example of these 4 classes are available as Supplementary Material.

### Traditional machine learning models

We deployed 3 traditional machine learning and deep learning models, that is, support vector machine (SVM),^35^^,^^36^ RF,^37^^,^^38^ and CNN,^39^^,^^40^ which were widely used in the previous studies and achieved good performance on the various NLP classification tasks. For the model developing, we employed the Word2Vec approach to convert the PPMs into numerical vectors, considering word relevance as the input for the traditional models.^41^ Subsequently, we utilized the scikit-learn package to implement the SVM and RF model, maintaining the default model configuration.^42^ The CNN model was developed using the Keras framework.^43^ We evaluated their performance on the multi-class classification of PPMs to identify patient primary concerns. The results provide a baseline for the performance comparison of pretrained BERT models. The detailed description of the applied traditional models is available as Supplementary Material.

### Pretrained BERT language models

In this study, we test 9 popular pretrained BERT models to build the baseline classifiers for PPMs triage. These 9 BERT models include 3 generic BERT models (BERT, RoBERTa, and ALBERT),^44–49^ 3 domain-specific models (BioBERT, BioClinicalBERT, and PubMedBERT),^50–54^ and 3 source-specific models (BERTweet, TWhinBERT, and RedditBERT).^55–58^ The detailed description of these pretrained BERT models and hyperparameter settings is available as Supplementary Material.

### Fusion framework design

**Figure 1.**
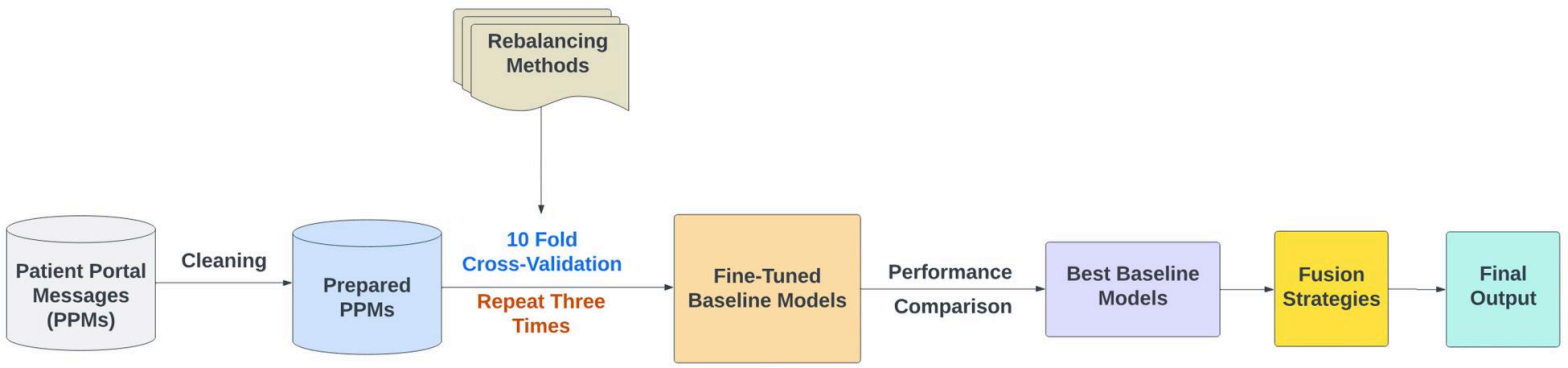
An overview of the fusion framework of pretrained language models for enhancing patient primary concern identification.

This study proposes a novel fusion framework which integrated the top-performing pertained LMs with a fusion layer to improve the performance on the multi-class classification of PPMs for patient primary concern recognition. The framework is shown in Figure 1, which includes data preparation, baseline model selection, fusion model optimization, and final model evaluation.

**Figure 2.**
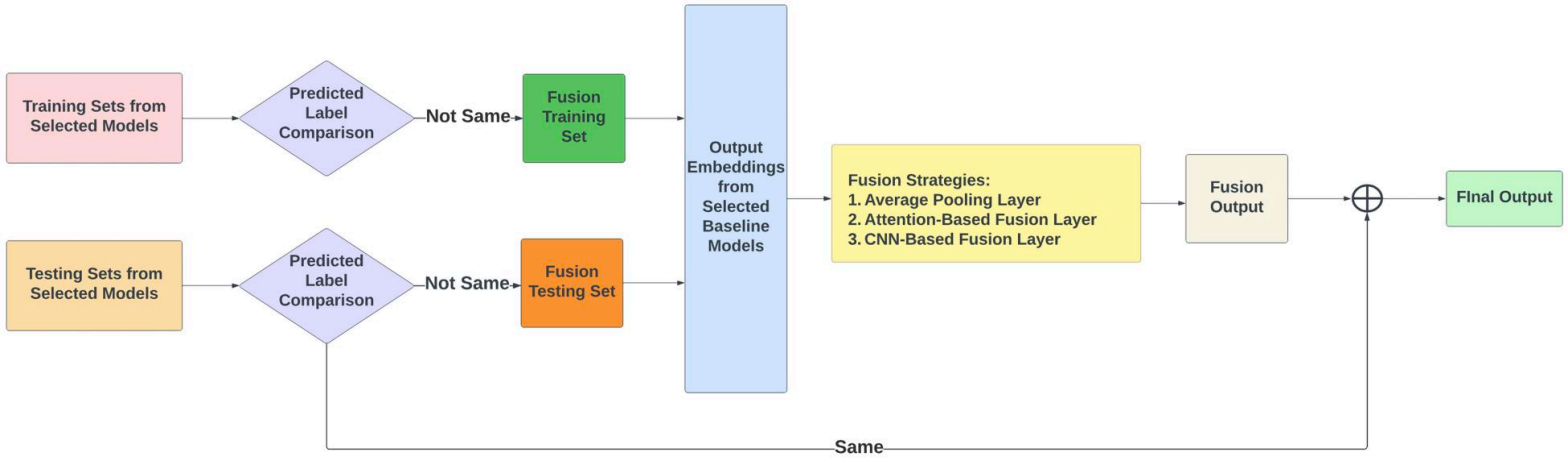
An overview of applied fusion strategies.

After fine-tuning baseline models, we identified the top-performing models within each LM category—generic, domain-specific, and source-specific—by considering their average performance metrics. We then applied a fusion approach to these chosen baseline models, as shown in Figure 2. We began by comparing the label predictions from the selected models for both the training and testing datasets. The PPMs with different predicted labels from the selected baseline models during training were used to formulate the training data by extracting the corresponding feature embeddings from the last hidden layer of the LMs, which were used for optimizing the fusion model. During the testing phase, we followed the same procedure as in the training phase. We selected messages with different predicted labels from the selected baseline models and created the testing set for the fusion model. The well-trained fusion model generates prediction results for this testing set of messages, which were then combined with the messages that had the same predicted labels from the baseline models. The final model performance was evaluated based on the combined results.

**Figure 3.**
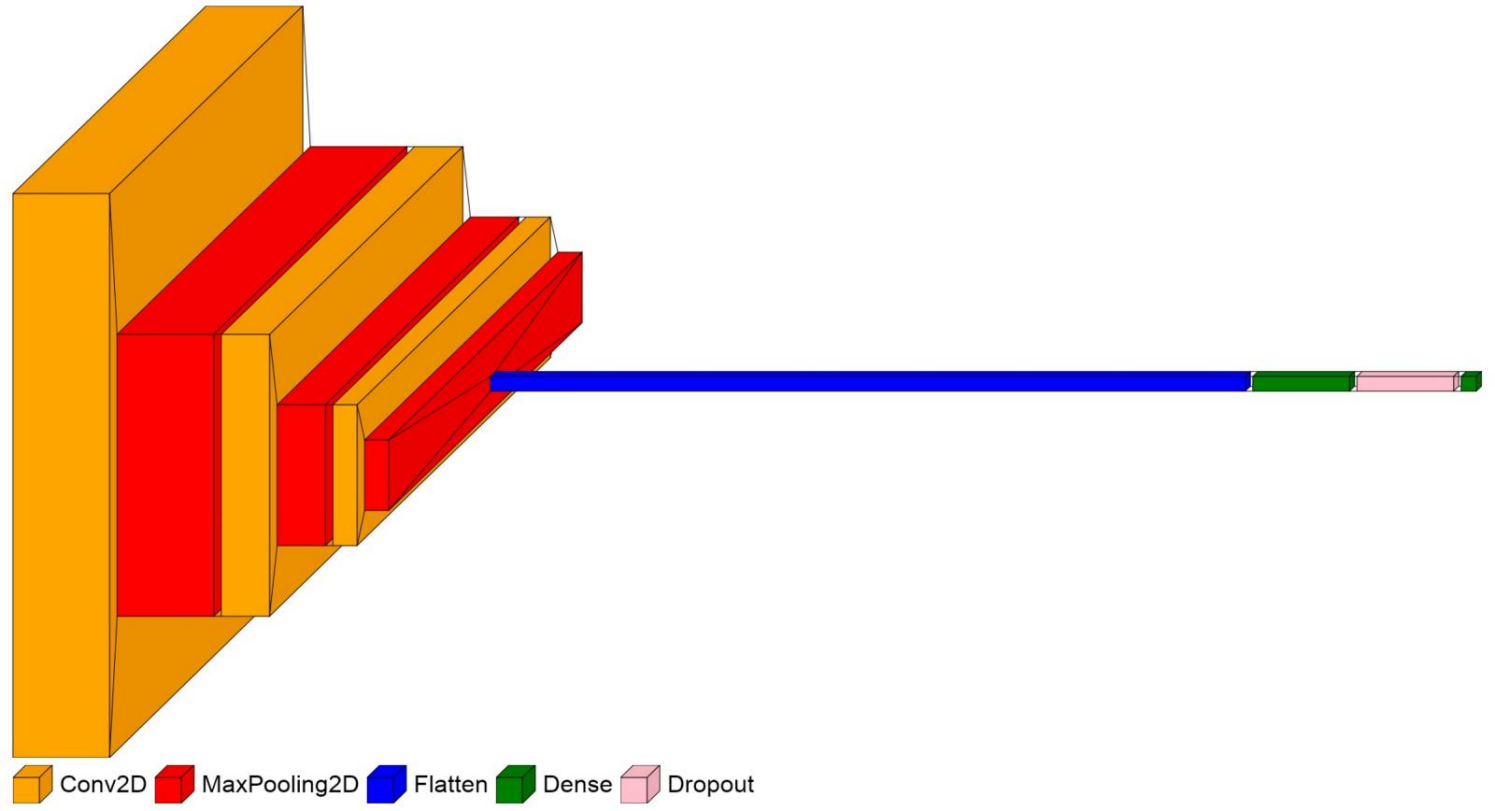
Architecture of CNN in the fusion framework.

As shown in Figure 2, we explored 3 different fusion strategies for elevating the performance of patient primary concern identification within PPMs. These fusion strategies include an average pooling layer, an attention-based fusion layer, and a CNN-based fusion layer. The fusion strategy *via* an average pooling layer involves computing the average values of the output embeddings from the selected pretrained LMs. Subsequently, a fully connected layer takes this average embedding to derive the final classification results. This method provides a straightforward integration of model output embeddings. The fusion strategy with an attention-based fusion layer assigns varying weights of importance to the output embeddings from the selected LMs by employing an attention mechanism. This prioritization allows for a more nuanced integration of model output embeddings, focusing on the most informative features for precise classification. The CNN-based fusion layer uses a 2D CNN model to efficiently synthesize the output embeddings of the selected LMs to enhance multi-class classification performance in PPMs. Figure 3 illustrates the architecture of the CNN model. The detailed description of the CNN model and hyperparameter settings is available as Supplementary Material.

We selected 3 top-performing baseline models—the best generic model, the best domain-specific model, and the best source-specific model. We implemented the fusion of the 3 baseline models *via* the 3 different fusion strategies—average pooling, attention mechanism, and CNN. We also explored the fusion of 2 baseline models (generic + domain, generic + source, and domain + source) *via* a CNN-based fusion layer. In addition, we compared the 6 fusion models with traditional ensemble models *via* majority voting. The majority voting approach serves as a foundational strategy and offers a straightforward and efficient consensus mechanism, providing a robust baseline for comparison. In cases where the 3 baseline models yield differing prediction labels where the majority voting fails to handle, the label chosen will be the one that comes first in alphabetical order: A, L, P, and U. We implemented 2 majority voting-based models by ensembling the 3 top-performing baseline models and the 3 fusion models of 2 baseline models *via* CNN. Table 1 listed the fusion and ensemble models implemented and evaluated in this study.

**Table 1.** The fusion and ensemble models implemented and evaluated in this study.

Fusion model name	Fusion layer	Embeddings from baseline models
3BERT + CNN	CNN	Generic, domain-specific, source-specific models
3BERT + Attn	Attention	Generic, domain-specific, source-specific models
3BERT + Aver	Average	Generic, domain-specific, source-specific models
2BERT(g, d) + CNN	CNN	Generic, domain-specific models
2BERT(g, s) + CNN	CNN	Generic, source-specific models
2BERT(d, s) + CNN	CNN	Domain-specific, source-specific models

Ensemble model name	Ensemble method	Baseline models
MV@3BERT	Majority vote	Generic, domain-specific, source-specific models
MV@3(2BERT + CNN)	Majority vote	2BERT(g, d) + CNN, 2BERT(g, s) + CNN, 2BERT(d, s) + CNN

### Experiments and evaluation

We implemented a 10-fold cross-validation approach to train and evaluate the 20 machine learning models in this study, including 3 traditional models, 9 BERT-based LMs, 6 fusion models, and 2 ensemble models. Each model underwent repeated cross-validation across every fold 3 times. This rigorous cross-validation process and the repetition of experiments are designed to guarantee the robustness and generalizability of each model across diverse datasets and experimental setups, thereby offering a more objective assessment of model performance.

To reduce the impacts posed by the imbalanced label distribution within our dataset, we introduced 3 rebalancing strategies during the training phase of each fold in our 10-fold cross-validation process. These strategies were designed to ensure a balanced representation of classes and enhance model performance across all labels. The first strategy involved applying balanced weights to adjust the importance of each class inversely proportional to its frequency. Our second strategy utilized random oversampling to augment the minority classes. Lastly, we employed a custom loss function that integrated focal loss, which places a greater focus on the correct classification of hard-to-learn examples. Each of these strategies was implemented with the goal of boosting the models’ abilities to recognize and accurately classify each label, particularly those less represented in the dataset.

The performance of each model was evaluated using accuracy, precision, recall, and *F*1 score, together with their mean values, standard deviation, and 95% confidence intervals.

## Results

**Figure 4.**
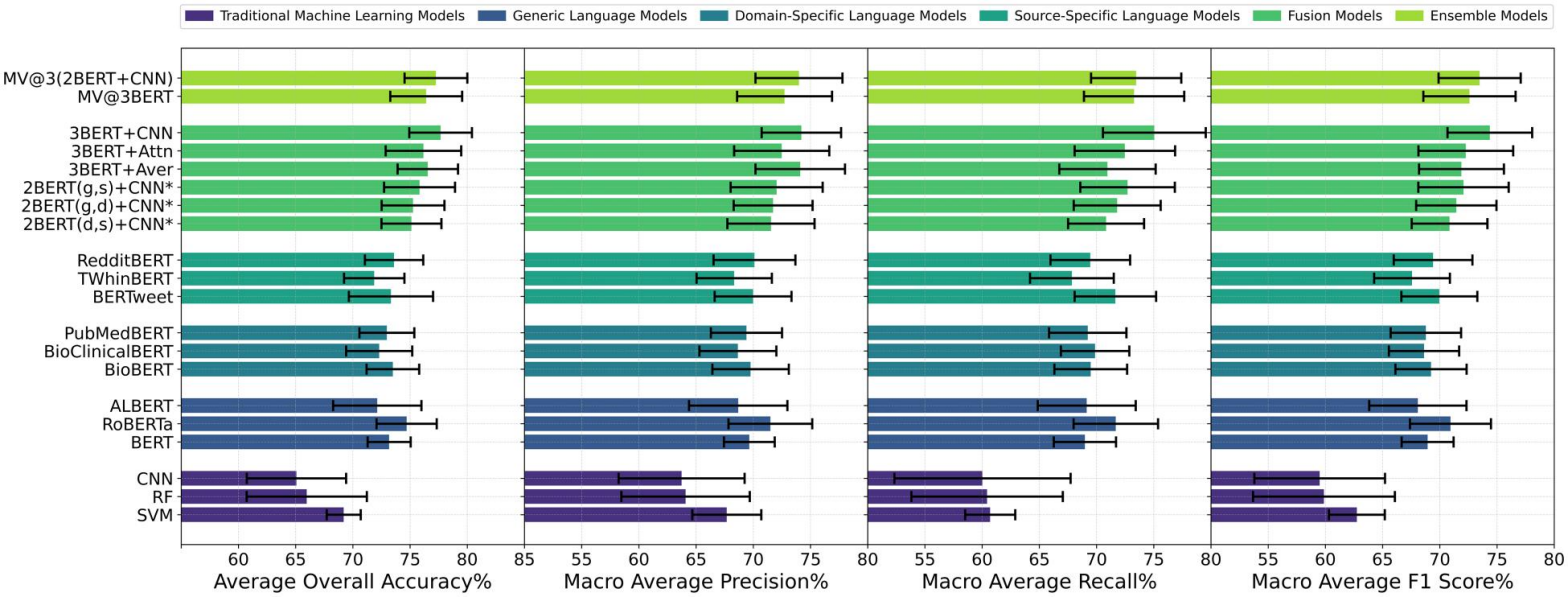
Comparison of model performance. *The g, d, and s denote generic, domain-specific, and source-specific language models, respectively.

We evaluated the performance of the 3 traditional machine learning models and the 9 pretrained BERT-based LMs to identify patient primary concerns for PPM triage. We also cross-compared the performance of our 6 fusion models and 2 ensemble models based on the 3 best baseline LMs. Figure 4 presents a comparative performance analysis of these 20 machine learning models in terms of overall average accuracy, macro-average precision, macro-average recall, macro-average *F*1-score, and the standard deviation of these measurements during the 10-fold cross-validation and the 3 repeated experiments for each fold. The detailed numbers of the performance measurements for all models are available as Table S2. Among the traditional machine learning models, SVM achieved the best performance in terms of an average overall accuracy of 69.20 ± 1.49% and a macro-average *F*1 score of 62.75 ± 2.44%, compared with RF and CNN. All the 9 pretrained BERT models had better performance than the traditional models. For instance, the original BERT model achieved an overall average accuracy of 73.17 ± 1.88% and a macro-average *F*1 score of 68.93 ± 2.27%. Among these BERT models, the best-performing model was the generic RoBERTa model, which achieved an average overall accuracy of 74.70 ± 2.63% and a macro-average *F*1 score of 70.93 ± 3.54%. For the domain-specific models, BioBERT demonstrated superior performance with an overall average accuracy of 73.50 ± 2.30% and a macro-average *F*1 score of 69.23 ± 3.11%. Among the source-specific models, BERTweet achieved the highest macro-average *F*1 score at 69.96 ± 3.32%, while RedditBERT recorded the best average overall accuracy of 73.60 ± 2.55%.

Based on the macro-average *F*1 scores of the baseline models, we selected the best generic model (RoBERTa), the best domain-specific model (BioBERT), and the best source-specific model (BERTweet) as 3 best baseline models to develop our fusion models, as shown in Figure 4. All the 6 fusion models we developed outperformed their corresponding baseline models. Among these fusion models, the combination of 3 output embeddings from the baseline models using a CNN model, 3BERT + CNN, was the most accurate, achieving an average overall accuracy of 77.67 ± 2.74% and a macro-average *F*1 score of 74.37 ± 3.70%. It exceeded the performance of both fusion models that merged 2 baseline models and those that integrated 3 baseline models utilizing alternative fusion strategies, including average pooling and attention. Among the 2 majority voting models, the model ensembling the 3 fusion models of 2 baseline models, MV@3(2BERT + CNN), reached a higher average overall accuracy of 77.26 ± 2.74% and a higher macro-average *F*1 score of 73.48 ± 3.59%, compared to the direct majority voting model of the 3 baseline models, MV@3BERT. However, both majority voting models were less effective than our top-performing fusion model, 3BERT + CNN that combined 3 BERT embeddings *via* CNN. This highlights the success of our fusion strategy for enhancing the identification of patient primary concerns in PPMs.

We also calculated precision, recall, and *F*1 score for each class (A, L, P, or U) across the original BERT model, the best baseline models (RoBERTa, BioBERT, and BERTweet) in each of the 3 LM categories, and the best fusion model (3BERT + CNN) as shown in Table 2. The results show all the 5 models demonstrated exceptional performance on identifying Active Symptom Concerns (Label A), achieving *F*1 scores exceeding 84% and standard deviation less than 2%. However, performance on other concerns or labels was significantly lower, with *F*1 scores ranging from 62% to 71%. Our fusion model achieved superior *F*1 scores across all 4 labels (A, P, L, and U) compared to these baseline models. Notably, it delivered a significant improvement of 6.32% in *F*1 score for label L, exceeding the gains of 2-3% observed for other concerns or labels. These results further underline the efficacy of our fusion approach in enhancing the model performance in the identification of patient primary concerns in PPMs.

**Table 2.** Comparison of the original BERT, the best baseline models, and our proposed fusion model.

Model	Label A			Label P		
	Precision%	Recall%	*F*1 score%	Precision%	Recall%	*F*1 score%
BERT	83.73 ± 3.85	85.77 ± 4.14	84.63 ± 1.85	63.80 ± 8.97	65.00 ± 10.10	63.77 ± 7.47
RoBERTa	85.27 ± 2.74	85.87 ± 3.32	85.40 ± 1.19	64.53 ± 9.82	**72.87 ± 10.40** ^a^	67.60 ± 7.21
BioBERT	84.37 ± 3.47	84.37 ± 3.95	84.10 ± 1.99	63.63 ± 9.29	65.50 ± 11.51	63.93 ± 8.81
BERTweet	85.17 ± 3.97	84.80 ± 4.12	84.63 ± 1.63	60.73 ± 5.47	72.80 ± 11.66	65.57 ± 5.59
3BERT + CNN	**87.16 ± 3.41** ^a^	**87.43 ± 3.29** ^a^	**87.23 ± 1.49** ^a^	**67.76 ± 7.32** ^a^	72.41 ± 9.24	**69.83 ± 6.87** ^a^

Model	Label L			Label U		
	Precision%	Recall%	*F*1 score%	Precision	Recall%	*F*1 score%
BERT	64.53 ± 6.74	61.67 ± 9.67	62.57 ± 5.99	66.53 ± 4.18	63.50 ± 6.07	64.77 ± 3.61
RoBERTa	66.43 ± 11.21	64.73 ± 11.64	64.83 ± 8.60	69.73 ± 5.03	63.27 ± 9.47	65.90 ± 5.60
BioBERT	63.73 ± 6.21	62.40 ± 10.22	62.63 ± 6.29	67.30 ± 5.22	65.70 ± 6.68	66.23 ± 3.86
BERTweet	62.33 ± 8.41	68.20 ± 12.75	64.33 ± 7.40	71.67 ± 5.94	60.77 ± 8.99	65.30 ± 5.93
3BERT + CNN	**69.59 ± 9.08** ^a^	**73.32 ± 13.94** ^a^	**71.15 ± 10.31** ^a^	**72.27 ± 4.75** ^a^	**67.02 ± 6.06** ^a^	**69.25 ± 4.18** ^a^

a Boldface indicates the highest score within each performance metric and category.

## Discussion

The effective triage of PPMs offers substantial benefits to healthcare services that both streamline care delivery and enhance patient experience.^59^ Efficient categorization of PPMs allows for improved workflow efficiency, ensuring judicious allocation of resources and prompt attention to patient inquiries.^13^^,^^60^ By allocating different message types to the most suitable staff—from administrative queries to clinical issues—this approach optimizes human resource utilization, ensuring each patient concern is addressed by the most qualified individual.^14^ Moreover, effective PPM triage is instrumental in enhancing patient care, as it aids in early detection of critical issues, such as adverse post-procedural events or the need for medication adjustments, thereby fostering improved health outcomes and patient satisfaction.^61^^,^^62^

Simultaneously, understanding the concerns communicated by patients through PPMs is vital for patient-centered care, fostering empathy and trust essential for successful healthcare interactions. It aids in crafting accurate diagnoses and effective treatments aligned with individual patient needs.^63^ Addressing patient concerns enhances patient satisfaction and engagement, encouraging adherence to treatments and follow-up appointments.^8^ Ultimately, the dual focus on triaging PPMs and uncovering patient concerns, especially primary concerns, not only elevates care quality but also leads to superior health outcomes, fostering a healthcare environment where patients feel genuinely heard and actively participate in their care journey.

Thus, in this study, we focused on identifying patient primary concerns in PPMs by utilizing and enhancing multi-class classification methods explored in our previous research for PPM triage.^18^ Prior studies from other groups have employed binary classification to detect the presence or absence of specific communication topics including patient concerns and needs within PPMs.^13–15^ This approach typically involves deploying a single binary classifier to detect a particular type of topic or concern, with multiple binary classifiers used to identify various topics or concerns within a PPM. However, the binary classification method falls short in identifying the primary concern of a patient, because binary classification fundamentally operates on a binary premise—identifying whether a feature is present or absent, without providing a direct mechanism to rank or prioritize concerns, although indirect and complicated approaches (eg, sequential application with priority ranking after detection of each concern) could be crafted with binary classification for primary concern identification. To address this limitation, we developed multi-class classification models, where each class corresponds to a distinct category of primary concern, based on our previous work on PPM triage.^18^ These models are specifically designed to directly determine the primary concern category of each message, moving beyond the binary distinction to a better understanding of patients’ immediate needs.

Additionally, we proposed a novel fusion framework of pretrained LMs, leveraging their advanced capabilities to enhance accuracy and performance in patient primary concern detection. We found that the top-performing generic model (RoBERTa), the leading domain-specific model (BioBERT), and the foremost source-specific models (BERTweet) all exceeded the performance of the original BERT model.

However, no existing pretrained LMs, including RoBERTa, have learned the effective language representation of domain knowledge and language style similar to PPMs.^18^ Thus, we introduced a fusion framework that combines the advantages of generic, domain-specific, and source-specific models for processing PPMs generated by patients. Specifically, we combined the output embeddings from the best-performing generic model RoBERTa, the best domain-specific model BioBERT, and the best source-specific model BERTweet and integrated them using different fusion strategies. The fusion model of the 3 baseline models *via* CNN, 3BERT + CNN, outperformed the other fusion models and majority voting models and achieved a significantly improved performance compared to the individual BERT models. This fusion strategy includes a correction mechanism based on CNN to improve the prediction of messages with different predicted labels from the 3 selected BERT models. Notably, 3BERT + CNN delivered the highest performance for each class (A, L, P, or U), especially for the label ‘L’, where the macro-average *F*1-scoresaw an improvement of 6.32%, surpassing the 2-3% improvements observed in other categories. The results show that this fusion strategy can effectively leverage the strengths of different baseline models to improve the overall classification performance. Although the fusion model outperforms single models in performance, fine-tuning multiple pretrained LMs requires extra effort. Our fusion model 3BERT + CNN, which integrates the best generic, domain-specific, and source-specific models and CNN, exceeds the performance of the original BERT model by 5.43%. However, this enhancement necessitates fine-tuning 4 baseline models, a process that, while not overly time-consuming, adds to the slight complexity of model development.

**Figure 5.**
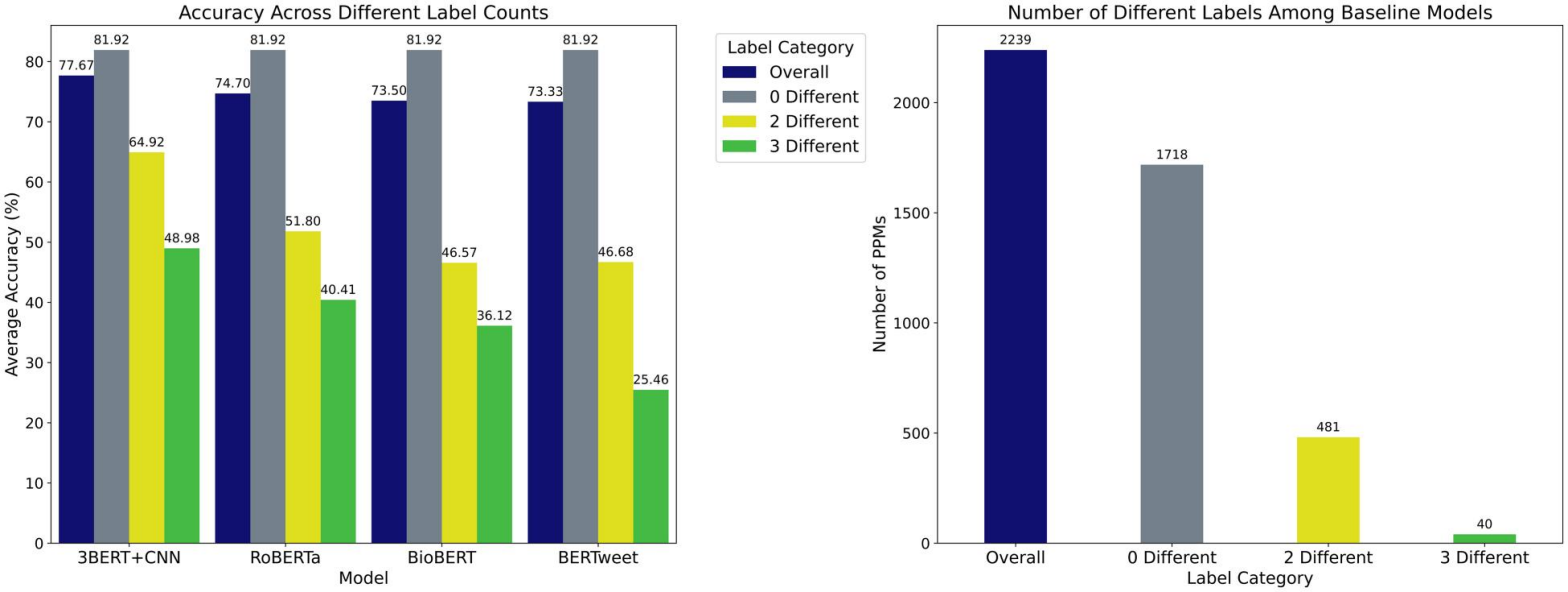
Analysis of performance enhancement of our fusion model from baseline models.

We analyzed the performance enhancement of our best fusion model, 3BERT + CNN, compared to the 3 baseline models (RoBERTa, BioBERT, and BERTweet) shown in the left subplot of Figure 5. We analyzed message classification for these baseline models and categorized them based on the number of distinct labels predicted by the 3 models (“0 different labels,” “2 different labels,” and “3 different labels”). In Figure 5, the right subplot shows the distribution of each label category. Our findings demonstrate significant improvements in classification accuracy for messages with discrepancies in label predictions from the baselines. Specifically, our fusion model achieved a 13.12% and 8.57% improvement in accuracy for messages with “2 different labels” and “3 different labels,” respectively. These results highlight our fusion model’s ability to effectively leverage the strengths of the individual baseline models, leading to an overall improvement in classification performance.

The success of the fusion framework suggests a feasible strategy to leverage existing pretrained LMs with different characteristics rather than pretraining specific LMs, which are time-consuming, for developing valid NLP algorithms for specific applications. Although the proposed fusion framework was demonstrated with an integrated model of 2-3 pretrained LMs including a generic LM, a domain-specific LM, and/or a source-specific LM, the fusion framework can also be used to combine other pretrained LMs. For example, the fusion model of multiple domain-specific LMs enables the investigation of the NLP tasks that require knowledge from different domains. The fusion model of BioClinicalBERT in the health domain and FinBERT^64^ in the financial domain would be valuable for studying cost-effectiveness in health care and other health economic problems. In the study, we developed and validated the fusion framework for PPM classification to identify patient primary concerns, but the fusion framework can be easily tuned for other text classification problems such as message priority and other NLP tasks such as named entity recognition (NER) because these NLP tasks are essentially categorized as classification problems in machine learning. In addition, the fusion framework of pretrained LMs can be used not only for analyzing PPMs generated by patients but also for mining patient narratives in other online health platforms including patient forums and social media.

While the fusion model has shown enhanced performance over individual pretrained BERT models, pinpointing the primary concerns in PPMs continues to be a formidable task. Patients often convey their primary concerns embedded within detailed background narratives. Consequently, PPMs are characteristically rich with multiple topics and concerns, which can obscure the primary issue that is of utmost importance to the patient. Table 3 lists 4 examples of misclassified PPMs as well as discussed topics and primary concerns. The primary focus of the first message revolves around the patient’s active eye symptoms. Although subsequent sentences in the message mention information about the appointment, the patient’s primary concern is not related to this topic. This is due to the patient having already scheduled an appointment and currently being more focus on the symptom relief. In the second message, although the patient described an active symptom, their primary concern lies in changing his or her primary care doctor, which falls under the logistic category. The first sentence in the third message outlines the current active symptom, while the last sentence reflects the patient's primary concern, which is seeking advice regarding medication prescription. The final message is an update after the patient's last visit. The patient has been consistently taking Metformin and observed a positive effect on their blood sugar levels. They expressed gratitude. This message includes the prescription-related topic, but it is not the patient’s primary concern. It is easily recognized as a prescription-related message by mistake due to the similar context. These sample messages demonstrated the challenges to detect patient primary concerns from multiple topics and concerns within the messages. Deciphering these primary concerns requires the model to skillfully navigate through a complex web of information presented in the messages.

**Table 3.** Examples, topics, and categories of misclassified Patient Portal Messages (PPMs).

Index	Deidentified and paraphrased message example	Included topics	Primary concern (label)	RoBERTa/BioBERT/BERTweet prediction	Fusion model prediction
1	My left eye missing eyelashes and flaky skin on the eyelid. I’ve just received the confirmation email and the documents upload request for the next week appointment. Do you have any additional suggestions to relieve it?	A, L	A	L	L
2	I saw my new primary physician. I have been having a pain in the middle of my abdomen for over 3 weeks. Dr. A ran examinations to find the problem. He arranged for a colonoscopy. I want to change my primary doctor to Dr. A, and no additional appointment needed	A, L	L	A	A
3	I’m experiencing the emergence of additional areas of hair loss on my scalp. Is it advisable to proceed with the treatment for these newly affected regions?	A, P	P	A	A
4	After the last visit, I’ve been consistently taking Metformin and have noticed that it’s truly helped to keep my blood sugar in the normal range. Thank you	U, P	U	P	P

While our current findings demonstrate significant advancements, further research is needed to understand more deeply the mechanism behind our fusion model’s performance, specifically on which factors and how they contributed to the model’s enhanced accuracy. Additionally, in forthcoming research, we intend to markedly enhance the performance to identify patient concerns amidst a myriad of topics within PPMs. We will incorporate tens of thousands of “PPMs” to expand the training set through data augmentation and/or weak supervision for refining the model. This strategic approach can minimize the need for labor-intensive and costly manual annotation processes. We would also consider the utilization of more expansive large LMs, incorporating cutting-edge generative AI technologies such as LLaMa, GPT, or Gemini^65–68^ and to adopt advanced strategies including prompt engineering, chain of thoughts, and knowledge injection.^69^ By leveraging these advanced methodologies, we anticipate not only distinguishing patient primary concerns with greater accuracy but also comprehensively capturing all the patient concerns from a range of topics and background information within the PPMs for enhancing the automatic PPM triage and patient concern recognition. However, using closed source large LMs, for example, GPT-4 to process patient health information requires careful consideration of legal and ethical guidelines to ensure patient privacy and confidentiality are protected. Additionally, the study was conducted using data from a single tertiary care institution in the United States. Regional dialects and the unique issues addressed at this single site may have influenced the content of the portal messages and, consequently, the performance of the classifier. To address these limitations, we plan to develop and validate our NLP algorithms for parsing PPMs across multiple healthcare sites.

## Conclusion

Efficiently triaging patient portal messages (PPMs) can greatly improve healthcare processes and ensure better allocation of resources. Highlighting patient concerns especially primary concerns in these communications not only emphasizes the importance of patient-centered care but also enhances the quality of overall healthcare by promptly acknowledging and addressing patient needs. In this study, we presented a novel fusion framework of pretrained language models (LMs) to develop multi-class classification algorithms for identifying patient primary concerns and triaging PPMs. We examined 3 traditional machine learning models (ie, SVM, RF, and CNN), 9 BERT-based LMs (ie, 3 generic models, 3 domain-specific models, and 3 source-specific models), 6 fusion models with 3 fusion strategies (ie, average pooling, attention mechanism, and CNN), and 2 ensemble models based on majority vote. Our results demonstrated that the proposed fusion model outperforms all the pretrained baseline models, achieving the highest performance for each class. This suggests that our framework successfully incorporates the features from different pretrained models, making it a promising solution to identity patient primary concerns in PPMs for PPM triage. This research offers a valuable contribution to text classification in the field of machine learning and NLP. It contributes meaningfully to the advancement of precise triage systems for PPMs and to a more nuanced comprehension of patient primary concerns, which is beneficial for patient-centered care.

## Supplementary Material

ocae144_Supplementary_Data

## Data Availability

The data underlying this article cannot be shared publicly due to the privacy of individuals that participated in the study.
